# Translation and validation of the Migraine Interictal Burden Scale (MIBS-4) in an Italian clinical cohort

**DOI:** 10.1007/s10072-026-09029-w

**Published:** 2026-04-17

**Authors:** Elisa Maria Piella, Alberto Mario Chiarandon, Maria Albanese, Enrica Maria Puddu, Francesca Pistoia, Gennaro Saporito, Marta Altieri, Giada Giuliani, Gloria Vaghi, Grazia Sances, Antonio Munafò, Alberto Chiarugi, Raffaele Ornello, Federico De Santis, Mariarosaria Valente, Renata Rao, Antonio Russo, Marcello Silvestro, Fausto Roveta, Danilo Antonio Montisano, Licia Grazzi, Stefania Battistini, Francesca Boscain, Edoardo Mampreso, Damiano Paolicelli, Simona Sacco, Cristina Tassorelli, Elisa Rubino, Maria Pia Prudenzano, Innocenzo Rainero

**Affiliations:** 1 Headache Center, Department of Neurosciences “Rita Levi Montalcini”, University of Turin, Turin, Italy; 2 Unit of Neurology, Department of Systems Medicine, University of Rome Tor Vergata, Rome, Italy; 3 IRCCS Neuromed, Pozzilli, IS Italy; 4 Department of Biotechnological and Applied Clinical Sciences, University of L’Aquila, L’Aquila, Italy; 5 Headache Center, San Salvatore Hospital, L’Aquila, Italy; 6 Department of Human Neurosciences, Sapienza University of Rome, Rome, Italy; 7 Department of Brain and Behavioral Sciences, University of Pavia, Pavia, Italy; 8 Headache Science and Neurorehabilitation Unit, IRCCS Mondino Foundation, Pavia, Italy; 9 Headache Center and Clinical Pharmacology Unit, Careggi University Hospital, Florence, Italy; 10 Department of Health Sciences, University of Florence, Florence, Italy; 11 DMED, University of Udine and Clinical Neurology, Udine University Hospital, Udine, Italy; 12 Neurology Unit, Department of Clinical and Experimental Sciences, University of Brescia, Piazzale Spedali Civili 1, 25123 Brescia, BS Italy; 13 Headache Centre, Department of Advanced Medical and Surgical Sciences, University of Campania “Luigi Vanvitelli”, Naples, Italy; 14 Neuroalgology Unit and Headache Center, Fondazione IRCCS Istituto Neurologico Carlo Besta, Milan, Italy; 15 Department of Medical, Surgical and Neurological Sciences, University of Siena, Siena, Italy; 16 Headache Centre, Neurology—Euganea, Health Unit, Padua, Italy; 17 “L. Amaducci” Neurological Clinics, Department of Translational Biomedicine and Neuroscience (DiBraiN), University of Bari, Bari, Italy

**Keywords:** Migraine, Burden, Interictal period, Validation, MIBS-4

## Abstract

**Background:**

The Migraine Interictal Burden Scale (MIBS-4) is a brief validated patient-reported measure of migraine interictal impact; however, an official Italian translation is not currently available.

**Methods:**

The MIBS-4 was translated and culturally adapted into Italian language and psychometrically evaluated in adults with episodic or chronic migraine enrolled in the Italian Headache Registry (RICe). In a 4-week test–retest design, participants completed the Italian version of MIBS-4 and the Migraine Disability Assessment (MIDAS) at baseline (T0) and at follow up (T1). We assessed internal consistency, dimensionality, test–retest reliability, convergent validity, and known-groups validity across MIDAS disability grades.

**Results:**

A total of 191 patients with migraine were included in test–retest analyses. Internal consistency was good to excellent (Cronbach’s α = 0.86 at T0; 0.90 at T1; ordinal α = 0.91–0.94; McDonald’s ω = 0.88–0.91). Parallel analysis supported a stable one-factor solution at both timepoints. Item-level agreement was moderate (weighted κ = 0.45–0.60; all *p* < 0.001), as was the total-score stability (ICC[A,1] = 0.62, 95% CI 0.50–0.72). Convergent validity with MIDAS was moderate (ρ = 0.40 at T0; ρ = 0.33 at T1; both *p* < 0.001).

**Discussion:**

The present findings indicate that the Italian MIBS-4 is psychometrically robust and captures a dimension of migraine burden that is only partially explained by conventional attack-focused disability measures.

**Conclusion:**

The Italian version of MIBS-4 provides a reliable instrument for quantifying interictal burden in Italian-speaking migraine individuals, enabling a more comprehensive evaluation of migraine and supporting individualized management.

**Supplementary Information:**

The online version contains supplementary material available at 10.1007/s10072-026-09029-w.

## Introduction

Migraine is a chronic neurovascular disorder characterized by recurrent headache attacks accompanied by nausea and/or vomiting, photophobia and phonophobia. It may present with, or be preceded by, transient focal neurological symptoms, known as aura [1]. The disease imposes a substantial burden on migraine individuals, families, and society, with the greatest impact observed among women of reproductive age [2].

Migraine is a cyclical disturbance of brain function comprising premonitory (including prodrome and aura), ictal, postdrome, and interictal phases, each involving distinct pathophysiological mechanisms and characteristic symptoms profile [3]. While the ictal phase of migraine has been extensively investigated, the interictal burden remains largely underexplored [4, 5]. Between attacks, patients may exhibit features such as allodynia, heightened sensory sensitivities, mood or cognitive changes, and reductions in health-related quality of life [4, 6]. Data from the Eurolight project indicates that 26% of individuals with migraine experience interictal symptoms, with 10.6% reporting anxiety associated with functional impairment. Similarly, the OVERCOME study showed that more than half of participants reported a meaningful burden even during headache-free intervals [7, 8]. A reduced sense of control over attacks can heighten interictal distress, particularly among individuals with migraine who have previously failed multiple preventive treatments. The fear of unforeseen attack fosters avoidance behaviors, including cephalalgiaphobia, which further amplify interictal disability and lower the threshold for acute medication use, increasing the risk of medication overuse [9, 10]. However, most clinical assessments of migraine severity remain centered on attack frequency and ictal disability, potentially overlooking a clinically relevant dimension of disease burden occurring between attacks. Therefore, assessing the interictal component of migraine is essential for comprehensive clinical management, which cannot be inferred from ictal measures alone.

Patient-reported outcome measures (PROMs) are invaluable in capturing the lived experience of migraine and quantifying its functional impact [11]. Among these, the Migraine Disability Assessment (MIDAS) is widely used to evaluate headache-related disability. Its reliability and simplicity have led to extensive clinical and research adoption [12]. MIDAS has been translated and validated in multiple languages, including Italian; however, by design, it captures only attack-related disability [13–16].

Despite its growing clinical relevance, only a limited number of validated instruments are currently available to quantify interictal burden [17]. To address this gap, Buse et al. developed the Migraine Interictal Burden Scale (MIBS-4), a brief four-item PROM that, to the best of our knowledge remains the only validated instrument specifically designed to quantify migraine interictal disability [5]. This instrument demonstrates moderate correlations with health-related quality of life and measures of ictal disability, supporting its use in both research and clinical practice [9].

This PROM was originally developed and validated in English, with subsequent translation in Portuguese [18]. However, no Italian translation or formal psychometric validation has been available to date, thereby limiting the assessment of interictal burden in Italian-speaking populations. Cultural and linguistic adaptation is critical in migraine research, as symptom expression and perceived disability may vary across sociocultural contexts [17]. This study aimed to validate an Italian version of the MIBS-4 as a reliable, conceptually equivalent, and clinically meaningful PROM for the assessment of interictal burden in migraine. Our final goal is to enhance multidimensional clinical evaluation and support more individualized treatment strategies that address both the ictal and interictal phases of the disease.

## Materials and methods

### Study design and ethics

In this prospective observational multicentre study, adult patients meeting ICHD-3 criteria for episodic (EM) or chronic migraine (CM) were recruited between July 2024 and February 2025. Participants were enrolled in the Italian Headache Registry (RICe). The local Ethics committee approved the study as part of the Registro Italiano Cefalee (RICe) study (Studio RICe, 14591_oss CEAVC Studio RICe, 14591_oss and subsequent amendments). Detailed information on the RICe study is reported in detail elsewhere [19]. All procedures were conducted in accordance with the Declaration of Helsinki and the European Union General Data Protection Regulation (GDPR) (EU 2016/679). Written informed consent was obtained from all participants prior to enrollment.

### Translation of the MIBS-4 questionnaire

Translation protocol followed international recommendations previously published [20]. The two translators responsible for the Italian adaptation were selected according to established criteria: both were native speakers of the target language, with one being a headache specialist and the other a bilingual professional with expertise in translation. The back-translation was subsequently performed by two independent bilingual translators with no prior knowledge of the original scale. All versions were reviewed by a multidisciplinary committee of translators and domain experts, who resolved discrepancies and ensured full lexical and cultural equivalence, producing the final Italian version of the instrument. The Italian translation of the questionnaire is available in Online Resource.

### Study population

To evaluate the applicability of the MIBS-4 questionnaire, we enrolled eligible patients with migraine with and without aura from 14 Italian tertiary headache centers. Eligibility criteria were: (i) age ≥ 18 years; (ii) diagnosis of episodic or chronic migraine according to ICHD-3 criteria; and (iii) clinical stability, defined as no changes in migraine preventive treatment during the study period. Patients were excluded if, in the judgment of the attending physician, they were unable to complete the questionnaire (e.g., cognitive impairment, illiteracy, or insufficient proficiency in Italian).

After obtaining written informed consent, participants completed an online survey administered via the Research Electronic Data Capture (REDCap) platform integrated within the RICe electronic data system. Participants completed the MIBS-4 and MIDAS questionnaires both at baseline (T0) and at 4-week follow-up (T1). Each patient received a unique identifier to link baseline and follow up assessments. Individuals with incomplete data on either questionnaire were excluded.

### Measures

In the present study, the translate version of the MIBS-4 and the validated Italian version of the MIDAS were administered. The MIBS-4 uses a 4-week recall period and comprises four items assessing functional impairment in work/study and family/social domains, difficulty planning or committing to activities, and emotional/affective and cognitive distress (Table 1) [9]. Item responses are summed to yield a total score ranging from 0 to 12, indicating: None (0), Mild (1–2), Moderate (3–4), Severe (≥ 5) interictal burden.

**Table 1:** Items included in the English version of the MIBS-4 questionnaire (adapted from Buse et al. [9])

Item	Don't know/NA	Never	Rarely	Some of the time	Much of the time	Most or all of the time
1. My headaches affect my work or school at times when I do not have a headache	□	□	□	□	□	□
2. I worry about planning social or leisure activities because I might have a headache	□	□	□	□	□	□
3. My headaches impact my life at times when I do not have a headache	□	□	□	□	□	□
4. At times when I do not have a headache, I feel helpless because of my headaches	□	□	□	□	□	□
Multiply number of checks by value = total score per column	*0	*0	*1	*2	*3	*3
Total score per column	____	____	____	____	____	____
Total score	Sum of column totals: ____________________________					

The MIDAS is a widely used PROM to quantify headache-related disability in both clinical practice and research. With a 3-month recall period, it captures disability as days of missed or markedly reduced productivity across three domains: paid work or school, household work, and non-work activities. The total score is obtained by summing responses to the five items, yielding a continuous measure (ranging from 0 to 270) that is commonly stratified into four disability grades: little/none (0–5), mild (6–10), moderate (11–20), severe (> 21). MIDAS has demonstrated high test–retest reliability and reasonably good validity [13].

### Statistical analysis

All statistical analyses were performed using R (version 4.5.1). Continuous variables were summarized using means and standard deviations, whereas categorical variables were reported as frequencies and percentages. The psychometric evaluation of the Italian MIBS-4 followed established recommendations for the validation of PROMs. Sample size adequacy was assessed according to COSMIN recommendations, which consider samples ≥ 100 participants adequate for studies of measurement properties [21].

Internal consistency was assessed at T0 and T1. Given the ordinal (0–3) response format of the items, reliability was evaluated using Cronbach’s alpha (α) and McDonald’s omega (ω) computed from polychoric correlation matrices. The dimensionality of the instrument was explored through Horn’s parallel analysis and exploratory factor analysis (EFA), conducted on both Pearson and polychoric correlation matrices. One-factor EFA models were then fitted at T0 and T1 using maximum-likelihood extraction.

Test–retest reliability over the 4-weeks interval was assessed in participants with complete MIBS-4 data at both timepoints. Item-level agreement between T0 and T1 was quantified using weighted Cohen’s κ with quadratic weights, whereas the stability of the total score was evaluated using the single-measure intraclass correlation coefficient (ICC, two-way mixed-effects model, absolute agreement). The magnitude of change between baseline and retest MIBS-4 total scores was quantified using Cohen’s *d* effect size calculated on paired observations. Agreement at the scale level was further explored through Bland–Altman analysis. Convergent validity was examined by correlating MIBS-4 total scores with MIDAS scores collected at corresponding timepoints using Spearman’s rank correlation coefficient. Known-groups validity was assessed by classifying participants according to standard MIDAS disability grades and comparing MIBS-4 scores across groups using the Kruskal–Wallis test. To assess potential attrition bias, baseline characteristics were compared between participants who completed the retest and those lost to follow-up using independent-samples t-tests for continuous variables and chi-square tests for categorical variables.

## Results

### Study populations characteristics

A total of 248 participants were enrolled in the study. Of these, 191 participants provided complete data at both timepoints and were included in the test–retest analyses (162 female, 84.4%; mean age 46.43 ± 13.43 years). Among these, only 32 participants were affected by Chronic Migraine (CM; 16.75%). The mean Monthly Migraine Days (MMD) was 8.20 ± 7.05, while the mean Monthly Medication Intake (MMI) was 7.81 ± 7.86. Migraine with aura was reported by 37 patients (20.7%) in the test–retest subsample. Demographic and clinical characteristics of the sample are summarized in Tables 2 and 3. The level of interictal disability was categorized into four classes according to the MIBS (Table 4).

**Table 2:** Demographic and clinical characteristics of the total sample and of the subgroup with valid MIBS-4 scores at both timepoints (T0-T1)

Variable	T0 sample (*N* = 248)	T0-T1 sample (*N* = 191)
Age (years)	45.36 ± 13.75	46.43 ± 13.43
Age at migraine onset (years)	18.78 ± 11.10	19.24 ± 11.38
Sex	216 (87.1%)	162 (84.8%)
Aura	43 (17.1%)	37 (20.7%)
MMD	8.31 ± 7.05	8.20 ± 7.05
MMI	7.98 ± 7.76	7.81 ± 7.86
MIDAS baseline	33.79 ± 43.47	34.10 ± 44.95
MIDAS retest	23.43 ± 30.70	23.15 ± 30.65
MIBS-4 T0	5.40 ± 3.93	5.40 ± 4.00
MIBS-4 T1	—	4.25 ± 3.71

Abbreviations: *MIBS-4* four-item migraine interictal burden scale, *MIDAS* migraine disability assessment scale, *MMD* monthly migraine days, *MMI* monthly medication intake

**Table 3:** Sex and age distribution across migraine frequency groups among participants with valid MIBS-4 at T1 (*N* = 191)

Migraine frequency group	*n*	Female *n* (%)	Age mean ± SD
Low-frequency episodic migraine	114	93 (81.6%)	46.1 ± 13.5
High-frequency episodic migraine	45	41 (91.1%)	46.0 ± 13.8
Chronic migraine	32	28 (87.5%)	48.1 ± 12.8

**Table 4:** Distribution of MIBS-4 disability grades at baseline (T0) and retest (T1)

MIBS-4 score	Level of interictal burden	T0 *n* (%)	T1* n* (%)
0	None	27 (14.1%)	38 (19.9%)
1–2	Mild	34 (17.8%)	42 (22.0%)
3–4	Moderate	28 (14.7%)	33 (17.3%)
5 +	Severe	102 (53.4%)	78 (40.8%)

### Internal consistency and factor structure

At baseline, the Italian MIBS-4 demonstrated a good internal consistency, with Cronbach’s α = 0.86 (95% CI: 0.83–0.89) and corrected item–total correlations ranging from 0.68 to 0.79. At T1, internal consistency increased to α = 0.90 (95% CI: 0.88–0.92), with item–total correlations between 0.72 and 0.91. Reliability estimates based on polychoric correlations yielded comparable results (ordinal α = 0.91 at T0 and 0.94 at T1), while McDonald’s ω further supported strong internal consistency (ω = 0.88 at T0; 0.91 at T1). Horn’s parallel analysis supported a one-factor solution at both timepoints using both Pearson and polychoric matrices, confirming the unidimensional structure of the Italian MIBS-4*.*

### Test–retest reliability

**Fig. 1:**
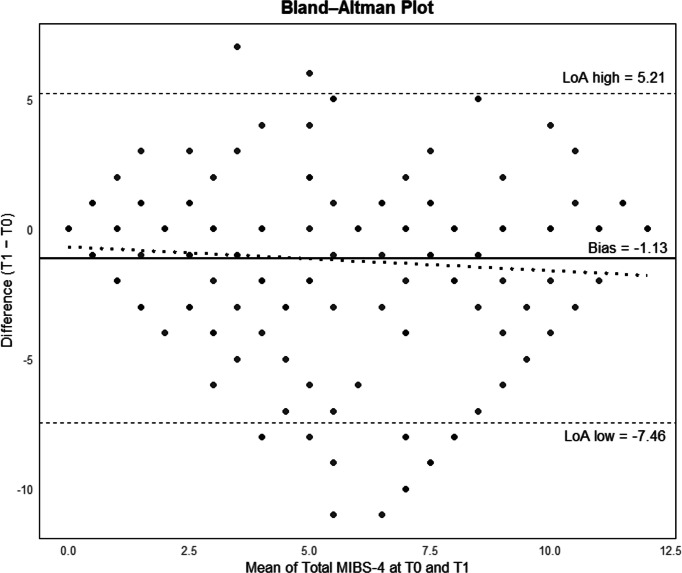
Bland–Altman plot showing agreement between MIBS-4 total scores at baseline (T0) and retest (T1). Dots represent individual participants plotted against the mean of T0 and T1 scores; the solid horizontal line indicates the mean difference (bias), and the dashed lines indicate the 95% limits of agreement (LoA)

Test–retest agreement was examined in the 191 participants with complete data. Weighted Cohen’s κ indicated moderate agreement for all items (κ = 0.60, 0.59, 0.56, and 0.45 for items 1–4; all p < 0.001). The stability of the total score was also moderate, with an ICC(A,1) of 0.62 (95% CI: 0.50–0.72). Mean MIBS-4 total scores varied from 5.40 ± 4.00 at baseline to 4.25 ± 3.71 at retest, corresponding to a small-to-moderate standardized mean change (Cohen’s d = − 0.35). The Bland–Altman plot showed a small negative mean difference (bias), with most observations falling within the 95% limits of agreement and no evidence of proportional bias across the range of total scores (Fig. 1).

### Convergent validity and known-groups validity

Convergent validity with migraine-related disability was examined by correlating MIBS-4 total scores with MIDAS scores. At baseline, MIBS-4 and MIDAS were moderately correlated (ρ = 0.40, *p* < 0.001). A similar correlation was observed at retest (ρ = 0.33, *p* < 0.001). Known-groups validity was supported by a clear gradient in MIBS-4 scores across standard MIDAS disability categories. At baseline, MIBS-4 scores differed significantly among minimal, mild, moderate, and severe MIDAS groups (Kruskal–Wallis χ^2^(3) = 32.51, *p* < 0.001), with higher interictal burden in the more impaired groups. A similar pattern emerged at retest (χ^2^(3) = 23.62*, p* < 0.001). MIBS-4 scores were also comparable between patients with and without aura at both baseline and retest. The dropout rate between baseline and retest was 23%. No significant differences were observed between completers and non-completers in baseline MIBS-4 scores (*p* = 0.99), MIDAS scores (*p* = 0.80), or sex distribution (*p* = 0.35), indicating limited risk of attrition bias.

## Discussion

In this prospective, observational, multicentre study, we evaluated the psychometric properties of the Italian version of the MIBS-4 in a clinical sample of patients with migraine. Overall, the findings support the reliability, unidimensional structure, and construct validity of the instrument, confirming its suitability for the assessment of interictal burden. The convergence of our findings with those of Ferrão et al. supports the cross-cultural robustness of the MIBS-4 [18]. The Italian version showed good internal consistency at both baseline and retest, with Cronbach’s α and McDonald’s ω values consistently exceeding recommended thresholds [21]. These results indicate that the four items coherently measure a single underlying construct, despite the brevity of the scale. Test–retest reliability analyses demonstrated moderate stability over the 4-week interval, as reflected by weighted Cohen’s kappa coefficients, and based on the intraclass correlation coefficient. Despite a small decrease in MIBS-4 scores at retest, stability indices indicated consistent measurement across time, supporting the reliability of the Italian MIBS-4. Bland–Altman analysis showed limited bias and no evidence of proportional bias, suggesting that this change likely reflects sensitivity to variations in interictal burden rather than measurement instability (Fig. 1).

Construct validity was further supported by moderate correlations with MIDAS scores, indicating that interictal burden is related to, but not redundant with, migraine-related disability [5]. In addition, known-groups validity analyses showed progressively higher MIBS-4 scores across increasing MIDAS disability categories, supporting the clinical relevance of the scale.

The translation and validation of this questionnaire provide clinicians with an additional instrument to assess the full impact of the disease on the patient and to support the migraine management, a condition increasingly recognized as a continuous disorder of brain function rather than a sequence of isolated pain episodes. Accumulating evidence indicates that patients frequently experience physical, cognitive, and affective symptoms between attacks, with a measurable impact on daily functioning [5, 22]. Consistently, neurophysiological studies have documented heightened cortical excitability between attacks, a plausible substrate for the sensory hypersensitivity that may persist even during headache-free intervals [23, 24].

Interictal symptoms have a substantial impact on patients’ quality of life and therefore warrant systematic assessment in routine clinical care. Despite increasing scientific interest, interictal disability remains underexplored in clinical practice, partly because of the limited availability of specific PROMs validated in the target languages. As a result, assessment often relies on monthly migraine days and ictal features, without capturing the overall clinical picture of the disease [5]. The MIBS-4 may serve as a practical decision-support tool, enabling individualized care by tailoring therapeutic strategies to the patient’s perceived overall disease burden.

From a clinical research perspective, it is worth highlighting that clinical guidelines recommend the use of disability measures as secondary outcomes to evaluate response to preventive treatments. However, a recent review of migraine clinical trials reported that only 40.3% included PROMs, underscoring the need for broader validation and cross-cultural adaptation of these instruments across languages [25].

The study benefited from a nationwide cohort recruited through specialized headache centers, where the higher level of expertise supports greater diagnostic accuracy. Nevertheless, several limitations should be acknowledged. Our sample was predominantly female (84.4%), a pattern that is common in headache centers but still limits generalizability to males. Most participants had episodic migraines, particularly low-frequency episodic migraine (59.7%), whereas chronic migraine accounted for only 16.8% of the sample. This imbalance in migraine subtypes may reduce the representativeness of the full clinical spectrum. Although this distribution is consistent with epidemiological data showing that episodic migraine is more prevalent than chronic migraine in the general population, it may nonetheless limit the representativeness of the full clinical spectrum, particularly for higher-burden patients.

A further point that warrants consideration is that the study sample was recruited from tertiary headache centres, potentially limiting the generalizability of the findings to non-specialist clinical settings; nevertheless, this context enhances the applicability of the results to clinical trial populations.

Even though the dropout rate between baseline and retest was 23%, no major differences emerged at baseline between those who completed follow-up and those who did not. Moreover, the available sample of 191 participants with complete test–retest data fulfils the threshold recommended by COSMIN for studies evaluating measurement properties [21].

The availability of a validated Italian version facilitates the integration of the MIBS-4 into routine practice to monitor interictal burden over time given its short length and apparent sensitivity to short-term changes. Its use encourages a more comprehensive assessment of migraine, capturing the broader and often underestimated consequences of the disorder between attacks. Future studies in more diverse samples and longitudinal designs specifically addressing responsiveness to treatment-related changes are warranted. This perspective is essential for aligning treatment strategies with the lived experience of patients.

## Conclusion

This study provides a validated Italian version of the MIBS-4, which to date is the only available validated questionnaire specifically designed to assess migraine-related interictal disability. This is particularly relevant because routine clinical evaluation, migraine evaluation is still largely driven by monthly migraine days, an approach that may underestimate the broader impact of the disease on patients’ lives. Its use may enhance clinical management by integrating interictal burden into patient assessment and improve outcome characterization in clinical trials. Additional studies in non-tertiary care settings and in samples with greater male representation is needed to further establish generalizability and external validity across broader, more heterogeneous populations.

## Supplementary Information

Below is the link to the electronic supplementary material.

Supplementary file1 (DOCX 17 KB)

## Data Availability

The data collected and analyzed for the current study are available from the corresponding author on reasonable request.
