# Supplementary material for: Translation and validation of the Migraine Interictal Burden Scale (MIBS-4) in an Italian clinical cohort
Source: Neurol Sci. 2026 Apr 17;47(5):430. doi: 10.1007/s10072-026-09029-w (PMC13086818; doi:10.1007/s10072-026-09029-w)
Supplement: Supplementary file 1 — Supplementary file1 (DOCX 17 KB) [file 10072_2026_9029_MOESM1_ESM.docx]

**Supplementary Materials**

**Title: Translation and validation of the Migraine Interictal Burden Scale (MIBS-4) in an Italian clinical cohort**

|  | NON SO | MAI | RARAMENTE | ALCUNE VOLTE | PER GRAN PARTE DEL TEMPO | PER LA MAGGIOR PARTE DEL TEMPO/TUTTO IL TEMPO |  |
| --- | --- | --- | --- | --- | --- | --- | --- |
| I miei mal di testa influenzano il lavoro o la scuola anche quando non ho mal di testa. |  |  |  |  |  |  |  |
| Mi preoccupa il fatto di dover programmare attività sociali o di svago perché potrei avere mal di testa. |  |  |  |  |  |  |  |
| Il mio mal di testa ha impatto sulla mia vita anche quando non ho mal di testa. |  |  |  |  |  |  |  |
| Quando non ho mal di testa, mi sento comunque impotente a causa del mio mal di testa. |  |  |  |  |  |  |  |
| Numero totale di “X” nella colonna |  |  |  |  |  |  |  |
| Moltiplicare il numero di “X” per il valore = punteggio totale per colonna | *0 | *0 | *1 | *2 | *3 | *3 |  |
| Punteggio totale per colonna |  |  |  |  |  |  |  |
| Punteggio totale | + | + | + | + | + | + |  |

**Supplementary table 1.** Italian translation of the MIBS-4 questionnaire
